# 3-Benzyl-2-sulfanyl­idene-1,3-thia­zolidin-4-one

**DOI:** 10.1107/S1600536810051548

**Published:** 2010-12-15

**Authors:** Durre Shahwar, M. Nawaz Tahir, Muhammad Asam Raza, Naeem Ahmad, Saherish Aslam

**Affiliations:** a Department of Chemistry, Government College University, Lahore, Pakistan; b University of Sargodha, Department of Physics, Sargodha, Pakistan

## Abstract

In the title compound, C_10_H_9_NOS_2_, the five-membered heterocyclic ring and the benzyl moiety are oriented at a dihedral angle of 77.25 (4)°. In the crystal, infinite polymeric *C*(6) chains extending along [001] are formed due to C—H⋯O hydrogen bonds. C—H⋯π inter­actions link the chains, building up a three-dimensional network.

## Related literature

For background to our inter­est in the sythesis of thia­zolidin derivatives and related structures, see: Shahwar *et al.* (2009*a* ▶,*b* ▶, 2010 ▶). For graph-set notation, see: Bernstein *et al.* (1995 ▶).
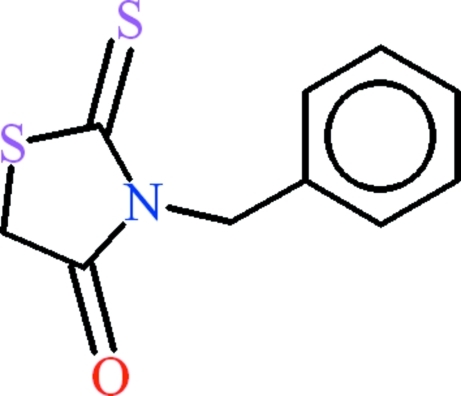


## Experimental

### 

#### Crystal data

C_10_H_9_NOS_2_
*M* *_r_* = 223.30
Monoclinic, 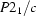

*a* = 13.3271 (4) Å
*b* = 5.9025 (2) Å
*c* = 13.0396 (4) Å
β = 92.812 (1)°
*V* = 1024.50 (6) Å^3^
*Z* = 4
Mo *K*α radiation
μ = 0.48 mm^−1^
*T* = 296 K
0.25 × 0.20 × 0.10 mm

#### Data collection

Bruker Kappa APEXII CCD diffractometer
Absorption correction: multi-scan (*SADABS*; Bruker, 2005 ▶) *T*_min_ = 0.939, *T*_max_ = 0.950
7899 measured reflections
1818 independent reflections
1594 reflections with *I* > 2σ(*I*)
*R*_int_ = 0.023

#### Refinement

*R*[*F* ^2^ > 2σ(*F* ^2^)] = 0.030
*wR*(*F* ^2^) = 0.079
*S* = 1.07
1818 reflections
127 parameters
H-atom parameters constrained
Δρ_max_ = 0.22 e Å^−3^
Δρ_min_ = −0.14 e Å^−3^

### 

Data collection: *APEX2* (Bruker, 2009 ▶); cell refinement: *SAINT* (Bruker, 2009 ▶); data reduction: *SAINT*; program(s) used to solve structure: *SHELXS97* (Sheldrick, 2008 ▶); program(s) used to refine structure: *SHELXL97* (Sheldrick, 2008 ▶); molecular graphics: *ORTEP-3 for Windows* (Farrugia, 1997 ▶) and *PLATON* (Spek, 2009 ▶); software used to prepare material for publication: *WinGX* (Farrugia, 1999 ▶) and *PLATON*.

## Supplementary Material

Crystal structure: contains datablocks global, I. DOI: 10.1107/S1600536810051548/dn2634sup1.cif

Structure factors: contains datablocks I. DOI: 10.1107/S1600536810051548/dn2634Isup2.hkl

Additional supplementary materials:  crystallographic information; 3D view; checkCIF report

## Figures and Tables

**Table 1:** Hydrogen-bond geometry (Å, °) *Cg* is the centroid of the C1–C6 ring.

*D*—H⋯*A*	*D*—H	H⋯*A*	*D*⋯*A*	*D*—H⋯*A*
C6—H6⋯O1^i^	0.93	2.47	3.338 (2)	156
C3—H3⋯*Cg*^ii^	0.93	2.95	3.674 (2)	136
C9—H9a⋯*Cg*^iii^	0.97	2.66	3.588 (2)	160

Symmetry codes: (i) 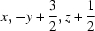; (ii) 
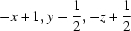
; (iii) 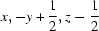.

**Fig. 1.**
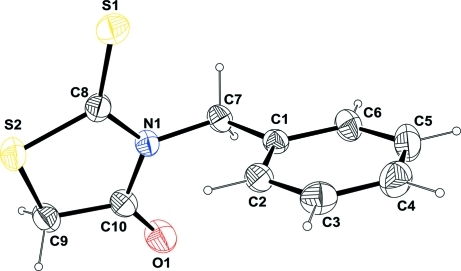
View of the title compound with the atom numbering scheme. Thermal ellipsoids are drawn at the 30% probability level. H-atoms are represented as small circles of arbitrary radii.

**Fig. 2.**
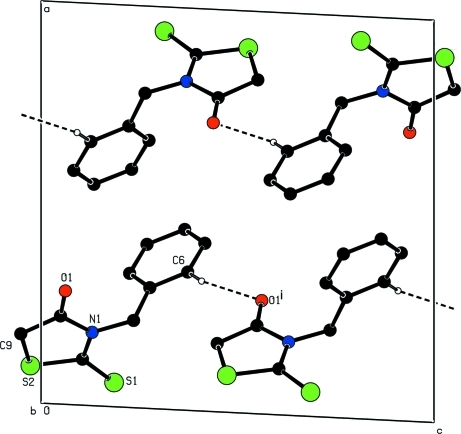
Partial packing showing the formation of the chains through C-H···O hydrogen bonds represented as dashed lines. H atoms not involved in hydrogen bondings have been omitted for clarity. [Symmetry codes: (i) x, -y+3/2, z+1/2]

## supplementary crystallographic information

### Comment

The work presented is part of our interest in synthesizing various thiazolidin derivatives and confirming their structures by *x*-ray analysis (Shahwar *et al.*, 2010, 2009**a*,b*). These compounds will be utilized for the study of comparative bioactivity.

In (I), the benzyl moiety A (C1—C7) and the five membered ring B (N1/C8/S2/C9/C10) of 2-thioxo-1,3-thiazolidin-4-one are planar with r. m. s. deviations of 0.0157 and 0.0302 Å, respectively. The dihedral angle between A/B is 77.25 (4)° (Fig. 1). In the 2-thioxo-1,3-thiazolidin-4-one, the attached O and S-atom are at a distance of -0.1070 (25) and 0.0763 (24) Å, respectively from the mean square plane of B.

Polymeric chains [C(6), Bernstein *et al.* (1995)] are formed due to C—H···O hydrogen bonds (Table 1, Fig. 2) and extend along the crystallographic *c* axis. C—H···π interactions (Table 1) link the chains to build up a three dimensional network.

### Experimental

The title compound has been prepared according to the method described (Shahwar *et al.* 2009**a*,b*)

### Refinement

All H-atoms were positioned geometrically (C–H = 0.93–0.97 Å) and treated as riding on their parent C atoms with *U*_iso_(H) = 1.2*U*_eq_(C).

### Crystal data

**Table:**

C_10_H_9_NOS_2_	*F*(000) = 464
*M**_r_* = 223.30	*D*_x_ = 1.448 Mg m^−^^3^
Monoclinic, *P*2_1_/*c*	Mo *K*α radiation, λ = 0.71073 Å
Hall symbol: -P 2ybc	Cell parameters from 1594 reflections
*a* = 13.3271 (4) Å	θ = 3.1–25.3°
*b* = 5.9025 (2) Å	µ = 0.48 mm^−^^1^
*c* = 13.0396 (4) Å	*T* = 296 K
β = 92.812 (1)°	Plate, light yellow
*V* = 1024.50 (6) Å^3^	0.25 × 0.20 × 0.10 mm
*Z* = 4	

### Data collection

**Table:**

Bruker Kappa APEXII CCD diffractometer	1818 independent reflections
Radiation source: fine-focus sealed tube	1594 reflections with *I* > 2σ(*I*)
graphite	*R*_int_ = 0.023
Detector resolution: 8.10 pixels mm^-1^	θ_max_ = 25.3°, θ_min_ = 3.1°
ω scans	*h* = −15→15
Absorption correction: multi-scan (*SADABS*; Bruker, 2005)	*k* = −6→7
*T*_min_ = 0.939, *T*_max_ = 0.950	*l* = −15→15
7899 measured reflections	

### Refinement

**Table:**

Refinement on *F*^2^	Primary atom site location: structure-invariant direct methods
Least-squares matrix: full	Secondary atom site location: difference Fourier map
*R*[*F*^2^ > 2σ(*F*^2^)] = 0.030	Hydrogen site location: inferred from neighbouring sites
*wR*(*F*^2^) = 0.079	H-atom parameters constrained
*S* = 1.07	*w* = 1/[σ^2^(*F*_o_^2^) + (0.031*P*)^2^ + 0.4046*P*] where *P* = (*F*_o_^2^ + 2*F*_c_^2^)/3
1818 reflections	(Δ/σ)_max_ < 0.001
127 parameters	Δρ_max_ = 0.22 e Å^−^^3^
0 restraints	Δρ_min_ = −0.14 e Å^−^^3^

### Special details

**Table:**

Geometry. Bond distances, angles *etc*. have been calculated using the rounded fractional coordinates. All su's are estimated from the variances of the (full) variance-covariance matrix. The cell e.s.d.'s are taken into account in the estimation of distances, angles and torsion angles
Refinement. Refinement of *F*^2^ against ALL reflections. The weighted *R*-factor *wR* and goodness of fit *S* are based on *F*^2^, conventional *R*-factors *R* are based on *F*, with *F* set to zero for negative *F*^2^. The threshold expression of *F*^2^ > σ(*F*^2^) is used only for calculating *R*-factors(gt) *etc*. and is not relevant to the choice of reflections for refinement. *R*-factors based on *F*^2^ are statistically about twice as large as those based on *F*, and *R*- factors based on ALL data will be even larger.

### Fractional atomic coordinates and isotropic or equivalent isotropic displacement parameters (Å^2^)

**Table:**

	*x*	*y*	*z*	*U*_iso_*/*U*_eq_	
S1	0.06040 (4)	0.13455 (10)	0.18585 (4)	0.0638 (2)	
S2	0.09221 (4)	0.28252 (9)	−0.02729 (3)	0.0557 (2)	
O1	0.28194 (12)	0.7373 (2)	0.06176 (11)	0.0697 (5)	
N1	0.18237 (9)	0.4684 (2)	0.12987 (9)	0.0396 (4)	
C1	0.30044 (11)	0.3660 (3)	0.27679 (11)	0.0376 (5)	
C2	0.33354 (12)	0.1781 (3)	0.22537 (13)	0.0443 (5)	
C3	0.40774 (14)	0.0402 (3)	0.26955 (15)	0.0565 (6)	
C4	0.44939 (15)	0.0897 (4)	0.36558 (16)	0.0632 (7)	
C5	0.41747 (15)	0.2765 (4)	0.41688 (15)	0.0625 (7)	
C6	0.34380 (14)	0.4147 (3)	0.37321 (13)	0.0509 (6)	
C7	0.21689 (12)	0.5173 (3)	0.23542 (12)	0.0424 (5)	
C8	0.11439 (12)	0.3010 (3)	0.10525 (13)	0.0437 (5)	
C9	0.16998 (14)	0.5229 (3)	−0.05169 (13)	0.0531 (6)	
C10	0.21917 (13)	0.5926 (3)	0.04939 (13)	0.0458 (5)	
H2	0.30574	0.14374	0.16042	0.0531*	
H3	0.42949	−0.08609	0.23425	0.0678*	
H4	0.49897	−0.00340	0.39547	0.0758*	
H5	0.44570	0.31047	0.48170	0.0749*	
H6	0.32296	0.54169	0.40864	0.0611*	
H7A	0.16040	0.50313	0.27915	0.0509*	
H7B	0.23972	0.67325	0.23923	0.0509*	
H9A	0.22040	0.48311	−0.09975	0.0637*	
H9B	0.12960	0.64606	−0.08070	0.0637*	

### Atomic displacement parameters (Å^2^)

**Table:**

	*U*^11^	*U*^22^	*U*^33^	*U*^12^	*U*^13^	*U*^23^
S1	0.0582 (3)	0.0707 (4)	0.0624 (3)	−0.0218 (3)	0.0027 (2)	0.0073 (3)
S2	0.0562 (3)	0.0648 (3)	0.0451 (3)	−0.0033 (2)	−0.0074 (2)	−0.0115 (2)
O1	0.0892 (10)	0.0615 (9)	0.0584 (8)	−0.0281 (8)	0.0049 (7)	0.0054 (7)
N1	0.0425 (7)	0.0405 (7)	0.0354 (7)	−0.0013 (6)	−0.0010 (5)	−0.0012 (5)
C1	0.0404 (8)	0.0379 (8)	0.0347 (8)	−0.0064 (7)	0.0044 (6)	−0.0013 (6)
C2	0.0463 (9)	0.0459 (9)	0.0407 (9)	0.0003 (7)	0.0033 (7)	−0.0057 (7)
C3	0.0555 (10)	0.0499 (11)	0.0648 (12)	0.0086 (9)	0.0100 (9)	−0.0004 (9)
C4	0.0541 (11)	0.0672 (13)	0.0675 (13)	0.0063 (10)	−0.0052 (9)	0.0162 (11)
C5	0.0660 (12)	0.0712 (13)	0.0483 (11)	−0.0066 (10)	−0.0159 (9)	0.0056 (10)
C6	0.0612 (11)	0.0503 (10)	0.0407 (9)	−0.0039 (8)	−0.0029 (8)	−0.0065 (8)
C7	0.0502 (9)	0.0407 (9)	0.0363 (8)	0.0017 (7)	0.0020 (7)	−0.0064 (7)
C8	0.0395 (8)	0.0464 (9)	0.0448 (9)	0.0005 (7)	−0.0018 (7)	−0.0047 (7)
C9	0.0587 (10)	0.0617 (12)	0.0387 (9)	0.0052 (9)	0.0020 (8)	0.0030 (8)
C10	0.0516 (9)	0.0426 (9)	0.0433 (9)	0.0018 (8)	0.0035 (7)	0.0014 (7)

### Geometric parameters (Å, °)

**Table:**

S1—C8	1.6315 (18)	C4—C5	1.368 (3)
S2—C8	1.7424 (17)	C5—C6	1.378 (3)
S2—C9	1.7947 (19)	C9—C10	1.501 (2)
O1—C10	1.201 (2)	C2—H2	0.9300
N1—C7	1.459 (2)	C3—H3	0.9300
N1—C8	1.368 (2)	C4—H4	0.9300
N1—C10	1.389 (2)	C5—H5	0.9300
C1—C2	1.380 (2)	C6—H6	0.9300
C1—C6	1.388 (2)	C7—H7A	0.9700
C1—C7	1.507 (2)	C7—H7B	0.9700
C2—C3	1.384 (2)	C9—H9A	0.9700
C3—C4	1.376 (3)	C9—H9B	0.9700
			
C8—S2—C9	93.15 (8)	C1—C2—H2	120.00
C7—N1—C8	122.61 (13)	C3—C2—H2	120.00
C7—N1—C10	120.12 (13)	C2—C3—H3	120.00
C8—N1—C10	117.27 (13)	C4—C3—H3	120.00
C2—C1—C6	118.55 (15)	C3—C4—H4	120.00
C2—C1—C7	123.43 (14)	C5—C4—H4	120.00
C6—C1—C7	117.98 (15)	C4—C5—H5	120.00
C1—C2—C3	120.62 (16)	C6—C5—H5	120.00
C2—C3—C4	120.13 (18)	C1—C6—H6	120.00
C3—C4—C5	119.66 (19)	C5—C6—H6	120.00
C4—C5—C6	120.49 (18)	N1—C7—H7A	109.00
C1—C6—C5	120.55 (17)	N1—C7—H7B	109.00
N1—C7—C1	114.46 (13)	C1—C7—H7A	109.00
S1—C8—S2	122.86 (10)	C1—C7—H7B	109.00
S1—C8—N1	126.28 (13)	H7A—C7—H7B	108.00
S2—C8—N1	110.86 (12)	S2—C9—H9A	110.00
S2—C9—C10	106.99 (12)	S2—C9—H9B	110.00
O1—C10—N1	122.91 (16)	C10—C9—H9A	110.00
O1—C10—C9	125.76 (16)	C10—C9—H9B	110.00
N1—C10—C9	111.33 (14)	H9A—C9—H9B	109.00
			
C9—S2—C8—S1	176.53 (12)	C6—C1—C2—C3	0.6 (2)
C9—S2—C8—N1	−4.19 (13)	C7—C1—C2—C3	−177.17 (16)
C8—S2—C9—C10	5.77 (13)	C2—C1—C6—C5	−0.7 (3)
C8—N1—C7—C1	82.66 (18)	C7—C1—C6—C5	177.15 (17)
C10—N1—C7—C1	−96.82 (17)	C2—C1—C7—N1	−8.0 (2)
C7—N1—C8—S1	0.9 (2)	C6—C1—C7—N1	174.18 (14)
C7—N1—C8—S2	−178.35 (11)	C1—C2—C3—C4	−0.1 (3)
C10—N1—C8—S1	−179.60 (13)	C2—C3—C4—C5	−0.4 (3)
C10—N1—C8—S2	1.15 (18)	C3—C4—C5—C6	0.2 (3)
C7—N1—C10—O1	2.5 (2)	C4—C5—C6—C1	0.4 (3)
C7—N1—C10—C9	−177.06 (14)	S2—C9—C10—O1	174.34 (16)
C8—N1—C10—O1	−177.01 (16)	S2—C9—C10—N1	−6.12 (17)
C8—N1—C10—C9	3.4 (2)		

### Hydrogen-bond geometry (Å, °)

**Table:**

Cg is the centroid of the C1–C6 ring.

**Table:**

*D*—H···*A*	*D*—H	H···*A*	*D*···*A*	*D*—H···*A*
C6—H6···O1^i^	0.93	2.47	3.338 (2)	156
C3—H3···Cg^ii^	0.93	2.95	3.674 (2)	136
C9—H9a···Cg^iii^	0.97	2.66	3.588 (2)	160

Symmetry codes: (i) *x*, −*y*+3/2, *z*+1/2; (ii) −*x*+1, *y*−1/2, −*z*+1/2; (iii) *x*, −*y*+1/2, *z*−1/2.
